# Semaglutide Exposure During Pregnancy: Results from the FAERS Database

**DOI:** 10.3390/ph19081249

**Published:** 2026-08-08

**Authors:** Alessia Zinzi, Mario Gaio, Annamaria Mascolo, Gorizio Pieretti, Mattia Cipriani, Joao Marcos Della Ragione, Rossana D’Amato, Francesco Rossi, Annalisa Capuano, Rosanna Ruggiero

**Affiliations:** 1 Department of Life Sciences, Health, and Health Professions, Link Campus University, 00198 Rome, Italy; a.zinzi@unilink.it (A.Z.); a.mascolo@unilink.it (A.M.); francesco.rossi@unilink.it (F.R.); r.ruggiero@unilink.it (R.R.); 2 Campania Regional Centre for Pharmacovigilance and Pharmacoepidemiology, 80138 Naples, Italy; mattia.cipriani@unicampania.it (M.C.); joaomarcos.dellaragione@unicampania.it (J.M.D.R.); annalisa.capuano@unicampania.it (A.C.); 3 Department of Experimental Medicine, Section of Pharmacology “L. Donatelli”, University of Campania “Luigi Vanvitelli”, 80138 Naples, Italy; 4 Plastic Surgery Unit, University of Campania “Luigi Vanvitelli”, 80138 Naples, Italy; gorizio.pieretti@unicampania.it; 5 Maternal and Child Care Unit, Local Health Authority (ASL), 84121 Salerno, Italy; rs.damato@aslsalerno.it

**Keywords:** semaglutide, glucagon-like peptide-1 receptor agonist, pregnancy outcomes, fetal disorders, FAERS, pharmacovigilance

## Abstract

**Background/Objectives**: The increased incidence of obesity and type 2 diabetes, along with the growing use of glucagon-like peptide-1 receptor agonists (GLP-1 RAs), particularly semaglutide, among women of childbearing age makes it especially important to assess their safety profile during pregnancy. **Methods**: A post-marketing study was conducted using reports from the U.S. FDA Adverse Event Reporting System (FAERS) database (2017–2025). Descriptive analyses focused on pregnancy outcomes and fetal/neonatal disorders. Disproportionality analyses assessed adverse event (AE) reporting by System Organ Class (SOC) and pregnancy-related AEs associated with semaglutide versus other medications. A Standardized MedDRA Query (SMQ)-based disproportionality analysis was also performed. **Results**: A total of 249 cases involving semaglutide use during pregnancy were identified, comprising 922 AEs. “Injury, poisoning and procedural complications” was the most frequently reported SOC, while abortion- and pregnancy loss-related events accounted for 27.3% of reported AEs. Compared with other medications, semaglutide showed higher reporting odds for “Pregnancy, puerperium and perinatal conditions” (ROR 1.59; 95% CI 1.37–1.86), “Injury, poisoning and procedural complications” (ROR 1.93; 95% CI 1.70–2.20), and gastrointestinal disorders (ROR 1.72; 95% CI 1.38–2.15), with no significant association for “Congenital, familial and genetic disorders” or “Reproductive system and breast disorders”. SMQ-based analysis showed increased reporting odds for “Termination of pregnancy and risk of abortion” (ROR 3.71; 95% CI 3.07–4.48). **Conclusions**: Disproportionate reporting was identified for pregnancy- and perinatal-related events, procedural/exposure-related complications, and pregnancy termination, with no evidence of a disproportionate congenital-anomaly signal. These findings should be interpreted cautiously and do not necessarily indicate a causal association.

## 1. Introduction

Glucagon-like peptide-1 receptor agonists (GLP-1 RAs) are widely used for the treatment of type 2 diabetes and the chronic management of obesity, providing additional cardiovascular, hepatic, and renal benefits [1,2,3,4,5,6]. Semaglutide belongs to this class of drugs and is available in multiple formulations for subcutaneous or oral administration. Its therapeutic effects derive from glucose-dependent stimulation of insulin secretion and from clinically meaningful weight loss, partially mediated by appetite suppression and increased satiety [7,8,9,10].

In recent years, a substantial increase in semaglutide prescriptions dispensed at retail pharmacies in the United States has been observed, likely due to its superior glycemic control, weight loss efficacy, and guideline-endorsed cardiorenal benefits [11,12]. The clinical use of semaglutide has seen an increase, especially among overweight or obese individuals with cardiometabolic comorbidities, adolescents aged 12 and older, and women of childbearing age who take it even before becoming pregnant [13,14,15].

The rapid global dissemination of semaglutide among women of reproductive age has raised novel concerns regarding their safety in sensitive physiological states such as pregnancy. In fact, current clinical guidelines recommend discontinuation prior to conception due to the potential reproductive toxicity [7,8,9,10,11,13]. These recommendations are largely based on preclinical evidence, in which exposure to GLP-1 RAs in animal models was associated with reduced fetal weight and growth, delayed ossification, skeletal and visceral anomalies, and increased embryonic loss [16].

Human data remains scarce. A recent review identified only six published case reports or case series, describing a total of seven unplanned pregnancies exposed to GLP-1 RAs from the preconception period up to 17 weeks of gestation [17]. In most cases, treatment was discontinued upon pregnancy recognition and reported pregnancy outcomes were largely reassuring; however, the available evidence is extremely limited. More recently, an observational, prospective, multicenter study found no statistically significant association between first-trimester exposure to GLP-1 RAs and the risk of major congenital malformations [18]. Lastly, according to a recent systematic review that included 36 human studies, periconceptional or first-trimester exposure to GLP-1 receptor agonists was not consistently linked to an increased risk of major congenital malformations, fetal growth restriction, stillbirth, or neonatal mortality. Preterm birth, prenatal weight gain, hypertensive diseases, gestational diabetes, and other maternal outcomes varied among trials and lacked a consistent safety signal. However, most of the research’s observational designs, the small number of exposed pregnancies, and the dearth of information on ongoing therapy during pregnancy continue to limit the amount of evidence that is currently available [19].

While these findings provide preliminary reassurance regarding inadvertent early exposure, they are insufficient to support intentional use during pregnancy. Furthermore, the limited number of exposed pregnancies and the lack of evidence on continued treatment during pregnancy underscore the need for complementary real-world pharmacovigilance studies to further characterize the safety profile of semaglutide.

Given the limited human evidence, which primarily concerns inadvertent gestational exposure, and considering that intentional use during pregnancy remains contraindicated, a high degree of clinical vigilance is warranted. This pharmacovigilance study aims to evaluate the safety profile of semaglutide following unintended exposure during pregnancy by characterizing maternal, fetal, and neonatal adverse events associated with GLP-1 RA exposure, thereby contributing to more robust real-world evidence to inform clinical decision-making for healthcare professionals and patients.

## 2. Results

### 2.1. Descriptive Results

**Figure 1:**
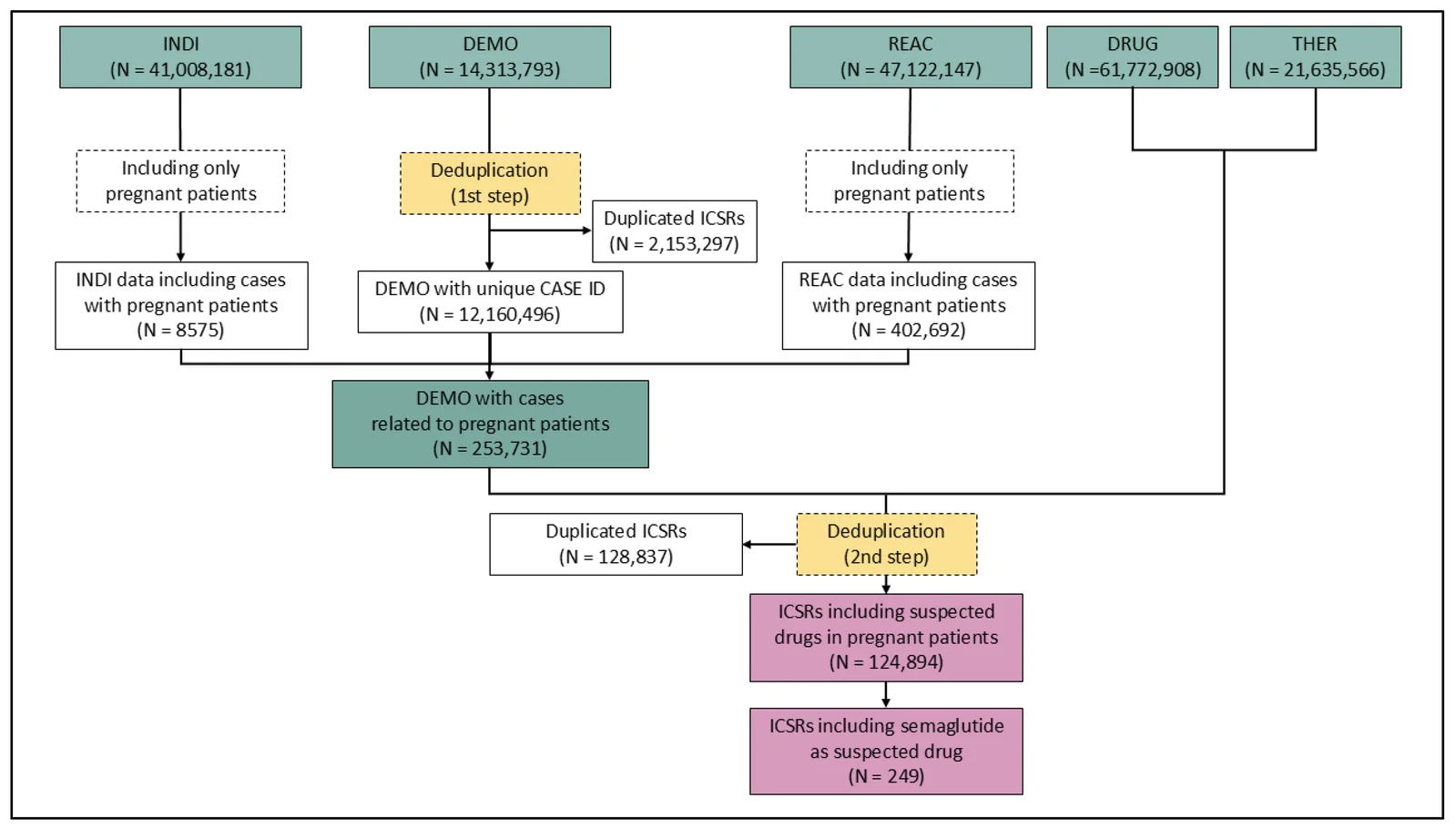
Flowchart for individual case safety reports (ICSRs) inclusion and exclusion criteria.

From 1 January 2017 to 30 June 2025, a total of 14,313,793 individual case safety reports (ICSRs) were retrieved from the FAERS database. Only 249 of the 124,894 incidents involving pregnant patients were linked to semaglutide (Figure 1).

The majority of ICSRs were submitted by consumers (*n* = 104; 41.8%) from the U.S. (*n* = 105, 42.2%), with a median reporter age of 34 years (*IQR*: 29–39 years). The indications for semaglutide were obesity (*n* = 120; 48.2%) and diabetes (*n* = 113; 45.4%), while in 16 cases (6.4%) the indication was not available. Regarding formulation, most reports involved an injectable product (*n* = 211, 84.8%), followed by oral semaglutide (*n* = 11, 4.4%); in 27 reports (10.8%), only the generic name “semaglutide” was recorded, precluding formulation identification. No case specified the exposure trimester in a structured PT or indication term. The median number of adverse events per ICSR was 3 (*IQR*: 2–4), and an increasing temporal trend in reporting was observed (Table 1).

**Figure 2:**
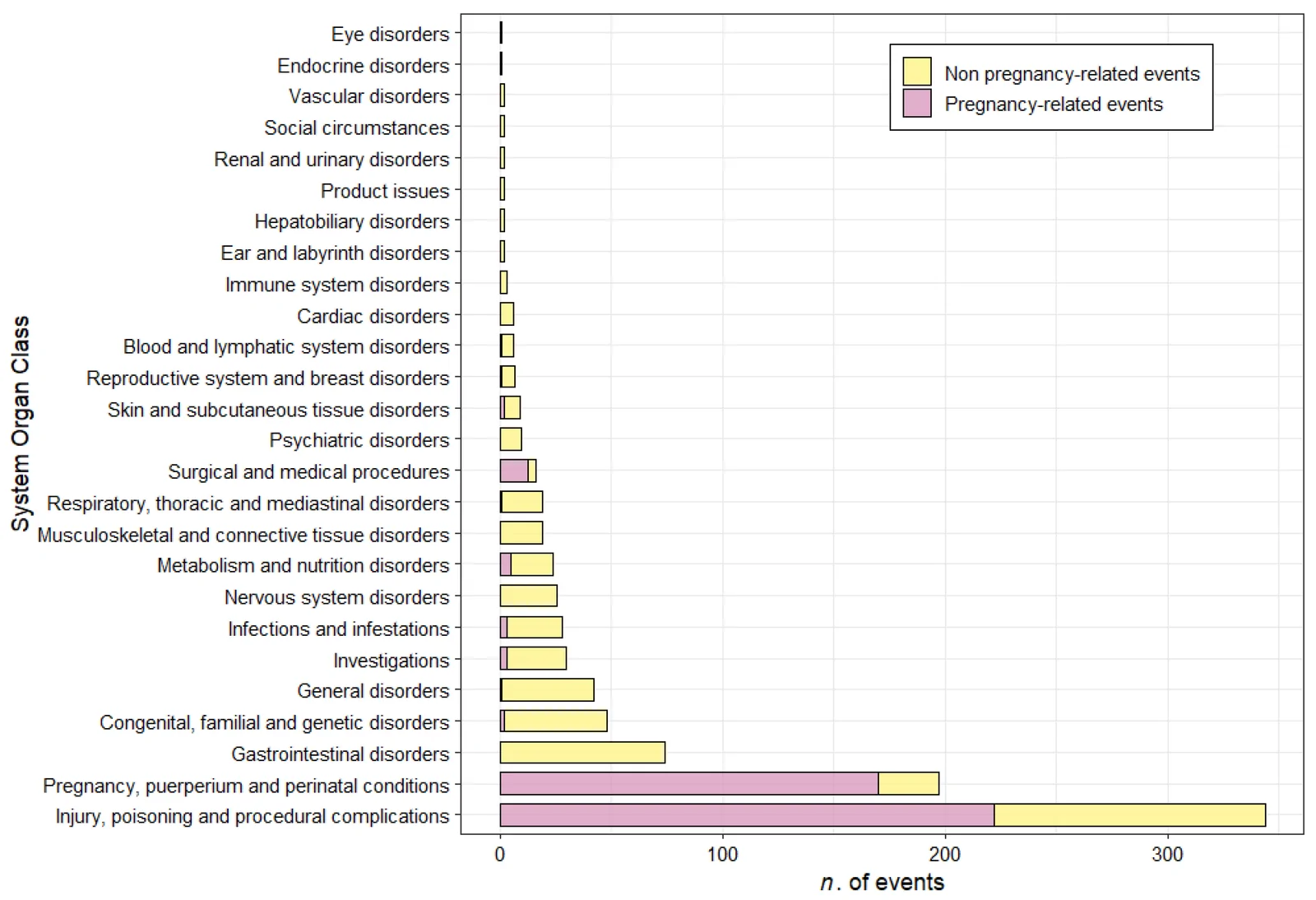
Frequency of the suspected adverse drug reactions according to their System Organ Class (SOC). Percentages were calculated using the total number of reported adverse events (*n* = 922) across all System Organ Classes as the denominator. Note: The high frequency of “Injury, poisoning and procedural complications” is largely driven by the preferred term “Maternal exposure during pregnancy”, an administrative exposure-documentation code required by MedDRA coding conventions for drug exposure during gestation, rather than an adverse clinical event.

Of the 922 reported events, more than one third was classified as “Injury, poisoning and procedural complications” (*n* = 344; 37.3%). The second most frequent system organ class (SOC) was “Pregnancy, puerperium and perinatal conditions” (*n* = 197; 21.4%), followed by “Gastrointestinal disorders” (*n* = 74; 8.0%), “Congenital, familial and genetic disorders” (*n* = 48; 5.2%), and “General disorders” (*n* = 42; 4.6%) (Figure 2).

Notably, the high frequency of “Injury, poisoning and procedural complications” can be attributed to the inclusion of the preferred term (PT) “Maternal exposure during pregnancy”, which was the most frequently reported pregnancy-related event (*n* = 162; 38.2%) (Table 2). Specifically, exposure-related events primarily reflected maternal or fetal exposure during or around pregnancy and included PTs such as “Foetal exposure during pregnancy” (*n* = 38), “Exposure during pregnancy” (*n* = 16), “Maternal drugs affecting foetus” (*n* = 2), “Maternal exposure before pregnancy” (*n* = 1), “Drug exposure before pregnancy” (*n* = 1), and “Maternal exposure timing unspecified” (*n* = 1). Overall, exposure-related events accounted for 221 reports (52.1%). Abortion- and pregnancy loss-related events constituted the second largest category (*n* = 116, 27.3%). Fetal and neonatal outcomes were reported less frequently (*n* = 36, 8.5%), while obstetric and maternal complications (*n* = 24, 5.7%) and delivery-related procedures or complications (*n* = 6, 1.4%) were comparatively uncommon (Table 2 reports only preferred terms (PTs) with more than two occurrences; PTs reported in only one or two cases are listed in Table S1).

### 2.2. Disproportionality Results

The disproportionality analysis comparing semaglutide with other medications in pregnant patients identified heterogeneous reporting patterns across SOCs (Table 3). Increased reporting odds were observed for “Pregnancy, puerperium and perinatal conditions” (ROR 1.59; 95% CI 1.37–1.86), “Injury, poisoning and procedural complications” (ROR 1.93; 95% CI 1.70–2.20), and “Gastrointestinal disorders” (ROR 1.72; 95% CI 1.38–2.15).

Conversely, decreased reporting odds were observed for “General disorders and administration site conditions” (ROR 0.40; 95% CI 0.29–0.54), “Psychiatric disorders” (ROR 0.30; 95% CI 0.16–0.54), “Skin and subcutaneous tissue disorders” (ROR 0.34; 95% CI 0.18–0.64), “Musculoskeletal and connective tissue disorders” (ROR 0.43; 95% CI 0.28–0.66), “Nervous system disorders” (ROR 0.66; 95% CI 0.47–0.93), and “Investigations” (ROR 0.67; 95% CI 0.47–0.97). No statistically significant differences were observed for the remaining SOCs, including “Blood and lymphatic system disorders” (ROR 0.58; 95% CI 0.26–1.31), “Cardiac disorders” (ROR 0.51; 95% CI 0.26–1.03), “Congenital, familial and genetic disorders” (ROR 1.22; 95% CI 0.91–1.62), “Infections and infestations” (ROR 0.78; 95% CI 0.55–1.12), “Metabolism and nutrition disorders” (ROR 1.25; 95% CI 0.83–1.87), “Reproductive system and breast disorders” (ROR 0.54; 95% CI 0.26–1.13), “Respiratory, thoracic and mediastinal disorders” (ROR 0.71; 95% CI 0.47–1.07), and “Surgical and medical procedures” (ROR 0.97; 95% CI 0.60–1.57).

The SMQ-based disproportionality analysis of pregnancy-related adverse events showed distinct reporting patterns for semaglutide compared with other medications (Table 4).

Lower reporting odds were observed for “Neonatal disorders” (ROR 0.37; 95% CI 0.24–0.56). No statistically significant disproportionality was identified for “Foetal disorders” (ROR 1.24; 95% CI 0.82–1.87). In contrast, increased reporting odds were observed for the SMQs “Pregnancy, labour and delivery complications and risk factors” (ROR 1.42; 95% CI 1.24–1.63) and “Termination of pregnancy and risk of abortion” (ROR 3.71; 95% CI 3.07–4.48).

Because the SMQ “Termination of pregnancy and risk of abortion” aggregates terms of heterogeneous clinical meaning, we further classified its constituent PTs as spontaneous pregnancy loss, elective/induced termination, or type not specified. The increased reporting odds were predominantly driven by spontaneous pregnancy loss (ROR 4.19; 95% CI 3.42–5.13), while a less precise signal was observed for elective/induced termination (ROR 2.55; 95% CI 1.44–4.50); no significant association was found for terms with unspecified type (ROR 1.81; 95% CI 0.68–4.84).

Subgroup analyses by formulation and indication showed that the increased reporting within the “Pregnancy, puerperium and perinatal conditions” SOC persisted across all four strata (injectable ROR 1.76, 95% CI 1.49–2.08; oral ROR 2.45, 95% CI 1.21–4.93; diabetes indication ROR 1.60, 95% CI 1.17–2.17; obesity/weight-control indication ROR 2.25, 95% CI 1.76–2.88), suggesting that this potential signal is not attributable to a single formulation or a single underlying metabolic condition. A similarly consistent, though less pronounced, gastrointestinal signal was observed in the injectable (ROR 1.40, 95% CI 1.07–1.83), oral (ROR 3.35, 95% CI 1.40–8.00) and diabetes (ROR 1.96, 95% CI 1.28–2.98) subgroups, but not in the obesity/weight-control subgroup (ROR 1.13, 95% CI 0.71–1.80).

Some divergent findings emerged. The ROR for “Injury, poisoning and procedural complications” was markedly elevated in the obesity/weight-control (ROR 3.10, 95% CI 2.48–3.86) and injectable (ROR 2.25, 95% CI 1.95–2.60) subgroups but not in the oral subgroup; review of the underlying preferred terms showed this signal to be driven predominantly by reports of off-label or unsupervised use rather than by physical injury. Conversely, we observed significantly reduced RORs for “General disorders and administration site conditions”, “Investigations”, and “Nervous system disorders” in the injectable and obesity/weight-control subgroups (Table 5).

When stratified by therapeutic indication, no statistically significant disproportionality for “Pregnancy, labour and delivery complications and risk factors” was observed among reports related to diabetes (ROR 1.32; 95% CI 0.99–1.75), whereas an increased reporting odds was identified among reports related to obesity or weight-control (ROR 2.03; 95% CI 1.62–2.55). Reports with a diabetes indication showed increased reporting odds for “Foetal disorders” (ROR 2.46; 95% CI 1.34–4.50) and “Termination of pregnancy and risk of abortion” (ROR 2.19; 95% CI 1.37–3.51), while reports with an obesity or weight-control indication showed lower reporting odds for “Neonatal disorders” (ROR 0.21; 95% CI 0.08–0.55) and higher reporting odds for “Termination of pregnancy and risk of abortion” (ROR 5.39; 95% CI 4.03–7.21) (Table 6).

When semaglutide was benchmarked against an indication-matched active comparator (other antidiabetic/anti-obesity drugs in pregnancy, *n* = 3807 ICSRs; Table 7), the signal for “Pregnancy, puerperium and perinatal conditions” was no longer observed (ROR 1.01; 95% CI 0.86–1.18, versus ROR 1.59; 95% CI 1.37–1.86 against all other drugs), and the SMQ “Pregnancy, labour and delivery complications and risk factors” reversed direction, showing significantly lower reporting odds (ROR 0.78; 95% CI 0.68–0.90, versus ROR 1.42; 95% CI 1.24–1.63 against all other drugs). In contrast, gastrointestinal disorders remained significantly increased and, if anything, more pronounced against this comparator (ROR 2.59; 95% CI 2.05–3.28, versus ROR 1.72; 95% CI 1.38–2.15), and the SMQ “Termination of pregnancy and risk of abortion” remained robustly elevated (ROR 4.19; 95% CI 3.41–5.17, versus ROR 3.71; 95% CI 3.07–4.48). Reporting of metabolism and nutrition disorders was significantly lower against the active comparator (ROR 0.46; 95% CI 0.31–0.70).

### 2.3. Sensitivity Analysis

The sensitivity analyses confirmed the primary findings. Restricting the analysis to ICSRs in which semaglutide was reported as the primary suspected drug yielded results consistent with the main analysis. Similarly, restricting the analysis to physician-reported ICSRs did not materially alter the findings. Among the ICSRs included in the primary analysis, only seven reports involved concomitant exposure to medications with established teratogenic potential (lisinopril, losartan, perindopril, ramipril, valsartan, vincristine, and warfarin).

## 3. Discussion

The expanding use of semaglutide among women of reproductive age poses clinically relevant questions regarding its safety during pregnancy. Although current clinical guidelines recommend discontinuation prior to conception, these recommendations are largely precautionary and primarily based on preclinical findings rather than robust clinical data [16,17,20]. Gestational exposures generally reflect treatments initiated before conception or before pregnancy recognition, rather than therapies started or intentionally continued during pregnancy. These conditions frequently coexist in women prescribed GLP-1 RA.

Also, the exclusion of pregnant women from clinical trials and the requirement for effective contraception during GLP-1 RA studies have further limited the availability of prospective safety evidence [21].

In preclinical studies, exposure to GLP-1 receptor agonists was associated with pregnancy loss and fetal abnormalities at clinically relevant exposures; however, the extent to which these effects may reflect direct drug-related toxicity versus secondary consequences of reduced maternal food intake remains uncertain [16].

In this context, post-marketing pharmacovigilance analyses represent an essential complementary source of evidence, enabling the exploration of potential safety signals in real-world settings where unintended exposure may occur before pregnancy is recognized [22,23].

Our analysis identified 249 ICSRs involving pregnant women exposed to semaglutide among 124,894 reports retrieved from the FAERS database. Most cases concerned women with a median age of 34 years. Although data on the average age of pregnant women exposed to semaglutide remain limited, this finding appears consistent with the available literature [18,24,25,26].

Over the study period, an overall increasing trend in the number of pregnancy-related reports was observed, with a progressive rise in submissions in the most recent years. Although fewer reports were recorded in 2025, this likely reflects the partial inclusion of data limited to the first two quarters of the year rather than a true decline in reporting frequency. The progressive increase in pregnancy-related reports observed over time is likely attributable, at least in part, to the marked expansion in the clinical use of semaglutide and other GLP-1 RAs in recent years. Several real-world studies have shown a marked increase in GLP-1 RA prescriptions in recent years, particularly for weight management indications and among women of reproductive age, with semaglutide showing one of the fastest growth rates following its approval for obesity treatment and the publication of cardiovascular outcome data [27,28,29]. Moreover, increased media attention and public awareness surrounding semaglutide for weight loss may have contributed to stimulated reporting in recent years.

In spontaneous reporting systems, healthcare professionals are typically approximately sevenfold more likely to report suspected ADRs than non-healthcare professionals [30]. In contrast, 41.8% of the ICSRs were submitted by patients in our analysis, highlighting a substantial contribution of consumer reporting within this specific context. This pattern likely reflects the increasing awareness and direct involvement of patients in adverse drug reaction reporting following regulatory changes that enable consumer submissions. Interestingly, a similar trend was observed in our previous pharmacovigilance study evaluating TNF-alpha inhibitors in pregnant women with psoriasis, where a considerable proportion of reports originated from patients as well [31]. As previously noted, pregnancy represents a particularly sensitive condition in which women may feel more vulnerable and therefore more empowered in recognizing and reporting events occurring after treatment exposure. While patient reporting may enhance signal detection, it may also introduce variability in clinical detail and diagnostic accuracy. Therefore, the relatively high proportion of patient-submitted reports should be considered when interpreting the findings, as differences in report completeness and medical confirmation may influence the characterization of pregnancy-related outcomes.

The median number of adverse events reported per ICSR was 3 (IQR: 2–4), accounting for a total of 922 reported events.

When adverse events were analyzed according to SOC, the most frequently reported category was “Injury, poisoning and procedural complications”. This finding was largely driven by the inclusion of the PT “Maternal exposure during pregnancy,” which was the most commonly reported term. Notably, this term does not represent an adverse drug reaction per se but rather reflects exposure documentation. Its prominence is consistent with MedDRA coding conventions, which recommend the systematic inclusion of pregnancy exposure terms in cases involving drug exposure during gestation [32].

Events related to abortion and pregnancy loss constituted the second largest category (27.3%) in our analysis. However, the interpretation of these findings requires careful consideration of potential confounding factors. A recent systematic review by Mandal et al., synthesizing available evidence on semaglutide exposure before and during pregnancy, reported that rates of spontaneous abortion, miscarriage, and preeclampsia were comparable to those observed in matched control groups with diabetes or obesity [33]. Similarly, Dao et al. did not observe a statistically significant increase in the risk of spontaneous abortion among semaglutide-exposed pregnancies compared with women with diabetes not treated with GLP-1 RAs or overweight women without diabetes, although a higher proportion of elective terminations was noted [18]. Some observational studies have reported an increased risk of preterm birth among semaglutide-exposed pregnancies. For example, Kolding et al. observed a higher odd of preterm delivery compared with non-exposed pregnancies [34]. However, preterm birth is strongly associated with maternal metabolic conditions such as obesity, pre-existing diabetes, gestational diabetes, and hypertensive disorders of pregnancy [35,36,37]. These conditions frequently coexist in women prescribed GLP-1 RAs and are themselves established risk factors for medically indicated or spontaneous preterm birth. Consequently, disentangling the contribution of the underlying maternal condition from that of the drug exposure is inherently difficult in observational studies and spontaneous reporting systems. As a result, any pregnancy-related safety signal should be interpreted in light of these baseline maternal risk factors rather than being directly attributed to semaglutide.

To directly address this issue, we performed a secondary disproportionality analysis benchmarking semaglutide against an indication-matched active comparator (i.e., other antidiabetic and anti-obesity drugs reported in pregnancy) rather than against all other medications. Under this more conservative comparison, the signal for “Pregnancy, puerperium and perinatal conditions” was no longer observed (ROR 1.01; 95% CI 0.86–1.18), and the SMQ “Pregnancy, labour and delivery complications and risk factors” reversed direction entirely, showing significantly lower reporting odds (ROR 0.78; 95% CI 0.68–0.90). These findings indicate that both associations, as detected against a broad comparator, are largely attributable to the underlying maternal metabolic condition rather than to semaglutide exposure itself, and are consistent with the findings reported by Mandal et al. and Dao et al. discussed above [18,33]. In contrast, gastrointestinal disorders and the SMQ “Termination of pregnancy and risk of abortion” remained significantly elevated against this active comparator (ROR 2.59; 95% CI 2.05–3.28, and ROR 4.19; 95% CI 3.41–5.17, respectively), suggesting that these two signals are less likely to be fully explained by confounding by indication and may warrant closer clinical attention. A modest, borderline-significant decrease was also observed for “Congenital, familial and genetic disorders” (ROR 0.74; 95% CI 0.55–0.99) against the active comparator, in the opposite direction from the non-significant finding of the main analysis; given its borderline nature, this result should be interpreted cautiously rather than as evidence of a protective effect. It should be noted that this active comparator, being defined by indication fields that are frequently incomplete in FAERS, likely under-ascertains the true population of diabetic/obese pregnant patients on other therapies, and residual confounding and reporting biases inherent to spontaneous reporting systems cannot be excluded.

Maternal obesity is a well-established risk factor for preterm birth; according to the literature, in pregnant women with obesity, the fat tissue releases pro-inflammatory cytokines like tumor necrosis factor alpha (TNF-α), interleukin-6 (IL-6), and C-reactive protein (CRP) [38]. These cytokines mess with placental blood vessel growth and nutrient exchange, increasing the risk of preeclampsia, fetal growth restriction, and preterm birth. Also, maternal obesity is increasingly recognized as a significant risk factor for miscarriages due to dysregulated inflammatory responses, impaired endometrial receptivity, and metabolic disorders. Studies have shown that a pro-inflammatory intrauterine environment, marked by high levels of TNF-α, IL-6, and CRP, negatively affects embryo implantation and placental development. Additionally, obesity-induced oxidative stress and mitochondrial dysfunction can lead to poor egg quality and chromosomal abnormalities, further increasing the risk of spontaneous miscarriage.

It is also known that pregnancies complicated by diabetes are associated with a high rate of miscarriage and premature birth [39,40]. According to the study conducted by Hedderson et al., there is a link between spontaneous preterm birth and high glucose levels in mothers. In fact, the risk of spontaneous preterm birth went up proportionally with glucose levels during pregnancy [41]. Also, according to the study conducted by Miao and colleagues, in diabetic women there is a combination of immune dysfunction, vascular-placental damage, and metabolic changes that contribute to an increased risk of miscarriage. These processes prevent proper implantation, hinder placenta formation, and cause early placental insufficiency [42]. Our disproportionality analysis comparing semaglutide with other medications reported in pregnant women revealed a higher reporting odds ratio for the SOCs “Pregnancy, puerperium and perinatal conditions” (ROR 1.59; 95% CI 1.37–1.86) and “Injury, poisoning and procedural complications” (ROR 1.93; 95% CI 1.70–2.20). No significant association was observed for the SOC “Congenital, familial and genetic disorders” (ROR 1.22; 95% CI 0.91–1.62) or “Reproductive system and breast disorders” (ROR 0.54; 95% CI 0.26–1.13). Finally, a higher reporting odds ratio was observed for the SMQ “Termination of pregnancy and risk of abortion” (ROR 3.71; 95% CI 3.07–4.48). Disaggregating the SMQ “Termination of pregnancy and risk of abortion” by clinical type clarified the nature of this signal. The association was predominantly driven by terms indicating spontaneous pregnancy loss (ROR 4.19; 95% CI 3.42–5.13), rather than by elective or medically induced termination (ROR 2.55; 95% CI 1.44–4.50), indicating that this signal more likely reflects an underlying fetal-risk association than a pattern of voluntary pregnancy decisions. Nonetheless, the increased reporting of elective/induced termination itself deserves cautious interpretation: it may reflect genuine clinical decision-making in the context of uncertainty regarding semaglutide’s fetal safety, rather than a direct consequence of drug exposure, and cannot be fully disentangled from reporting or detection bias in a spontaneous reporting system.

Notably, the absence of a disproportionate congenital-anomaly signal in our analysis contrasts with a systematic review by Muller et al., who reported a higher incidence of significant congenital anomalies, decreased fetal weight and growth, delayed ossification, irregular ossification patterns, and skeletal variations [16]. However, that review was based on studies conducted in mice, rats, and rabbits, highlighting important gaps due to the lack of robust clinical data. Our finding is instead more consistent with a systematic review restricted to large human populations found no statistically significant differences in pregnancy outcomes between GLP-1 RA–exposed and control groups, including fetal growth restriction, small for gestational age, live births, major congenital anomalies, miscarriage, preterm birth, or stillbirth [43]. Potential pathophysiological mechanisms underlying pregnancy-related ADRs associated with semaglutide and other GLP-1 receptor agonists remain incompletely understood. Proposed mechanisms include reduced maternal caloric intake and weight loss resulting in impaired fetal nutrient supply and fetal growth restriction, altered placental development and nutrient transport, endocrine and reproductive alterations involving the hypothalamic–pituitary–gonadal axis, and embryofetal toxicity observed in animal studies, including delayed ossification and skeletal variations [16,44]. However, the available evidence remains limited, and several questions regarding the clinical relevance and translational applicability of these findings in humans are still unresolved.

The divergence between our findings and the largely reassuring results of large prospective cohort and systematic-review studies of GLP-1 receptor agonists in pregnancy warrants explicit methodological reconciliation. Spontaneous reporting databases such as FAERS are inherently prone to reporting biases that can generate disproportionality signals in the absence of any true increase in incidence. First, notoriety bias may be particularly relevant for semaglutide: its rapid uptake for weight management and extensive media coverage likely stimulated heightened reporting of adverse pregnancy outcomes among exposed women, an effect largely absent for the long-established comparator drugs. Second, FAERS captures a self-selected, non-random subset of exposed pregnancies, in which severe, unusual, or clinically salient outcomes are more likely to be reported, whereas prospective cohorts systematically ascertain all outcomes, including uneventful pregnancies, within a defined exposed population. Third, disproportionality analysis lacks a true denominator of drug exposure and cannot estimate incidence: an elevated ROR reflects a relative excess of reporting, which can arise from differences in reporting completeness or diagnostic scrutiny between drugs, not necessarily a true difference in event rates. Finally, unlike prospective cohorts, which can adjust for measured confounders by design or statistical modeling, disproportionality analysis affords only limited control for confounding by indication, as illustrated by our own active-comparator analysis. For these reasons, findings from disproportionality analyses such as ours should be regarded as hypothesis-generating signals for further investigation, complementary to rather than in direct conflict with the more reassuring evidence from prospective cohort and systematic-review studies; discrepant results should prompt closer scrutiny in adequately powered, prospectively designed studies rather than being interpreted as contradictory.

With regard to non–pregnancy-related adverse events, reporting of gastrointestinal disorders was disproportionately higher among pregnant women exposed to semaglutide (ROR 1.72; 95% CI 1.38–2.15) compared with pregnant women treated with medications other than semaglutide. Conversely, reporting of psychiatric disorders (ROR 0.30, 95% CI 0.16–0.54) and skin and subcutaneous tissue disorders (ROR 0.34; 95% CI 0.18–0.64) was significantly lower, while no significant association emerged for respiratory, thoracic and mediastinal disorders (ROR 0.71; 95% CI 0.47–1.07) or events related to surgical and medical procedures (ROR 0.97; 95% CI 0.60–1.57). The reduced reporting observed for several SOCs likely reflects the relative over-representation of pregnancy-related and gastrointestinal terms among semaglutide reports (i.e., a masking effect intrinsic to disproportionality analysis) rather than a genuine protective association, and should not be interpreted as such [45,46].

Consistent with the Summaries of Product Characteristics (SmPCs) and previous studies, gastrointestinal disorders were among the most frequently reported AEs [20,47]. Specifically, clinical studies report that 39.1–41.9% of patients experience gastrointestinal symptoms, mainly nausea (11.4–23.2%), in line with our analysis [48]. The reduced reporting of psychiatric and skin and subcutaneous tissue disorders, discussed above, is more consistent with a masking effect than with a true protective effect.

In this context, our findings should be interpreted cautiously. Given the intrinsic limitations of disproportionality analyses (particularly the absence of denominator data and the inability to adjust for underlying maternal comorbidities), these signals should not be interpreted as evidence of a causal association.

### Strengths and Limitations

Our study is based on FAERS, one of the largest spontaneous reporting systems worldwide, which allows the identification of potential safety signals in populations typically excluded from clinical trials, such as pregnant women. The use of standardized MedDRA terminology and disproportionality analysis enabled a structured assessment of pregnancy-related reporting patterns associated with semaglutide. In fact, the rationale for pharmacovigilance is based on the need to address the limits of pre-marketing trials: small sample sizes, limited duration, and the exclusion or the underrepresentation of specific population groups [49,50]. To our knowledge, this represents one of the largest real-world case series of semaglutide-exposed pregnancies reported to date, offering more precise estimates than the case reports and small case series that have so far characterized this population. Our analytical framework further combined a broad System Organ Class (SOC)-level disproportionality analysis with a complementary, pregnancy-specific Standardized MedDRA Query (SMQ)-based analysis, allowing detection of both generalized safety signals and more granular, clinically focused pregnancy and fetal/neonatal outcomes. However, important limitations must be acknowledged. The identification of pregnancy cases in spontaneous reporting databases is methodologically challenging, and misclassification or incomplete capture of relevant reports cannot be excluded. Moreover, our results are subject to typical biases encountered in pharmacovigilance, including under-reporting (only some of the adverse events observed in clinical practice are collected in pharmacovigilance databases), the tendency for serious events to be reported more frequently than non-serious events, and notoriety bias (the spontaneous reporting may be influenced in unknown ways by safety alerts; for example, important information notes from regulatory agencies, media attention, or scientific research can increase and trigger the reporting of specific adverse events regardless of any real change in risk.) [51]. Another limitation is that the FAERS database does not systematically capture gestational age or trimester of exposure in structured fields. As a result, stratification of cases according to the timing of semaglutide exposure during pregnancy was not feasible. Because the teratogenic risk of many medications is highly dependent on the stage of fetal development, the inability to evaluate trimester-specific patterns may have masked potential differences in the occurrence of pregnancy-related adverse outcomes. Moreover, spontaneous reports sent by patients can often be unclear, incomplete, and of low quality. In fact, the lack of information about gestational age, the duration of exposure to the suspected drug, the dosage used, any concomitant medications taken, comorbidities, and pregnancy outcomes can lead to misclassification of cases and later limit the assessment of causality. Detailed clinical and laboratory parameters relevant to disease severity, such as glycemic control or degree of obesity, are similarly unavailable, precluding adjustment for the severity of the underlying metabolic condition. Additionally, disproportionality analyses do not provide incidence estimates and cannot adjust for confounding factors. The denominators for drug exposure are not known, which makes it impossible to estimate incidence rates or absolute risks and limits comparisons between exposed and unexposed populations. For this reason, disproportionality analyses are generally used to generate potential safety signals rather than to establish causal associations or measure risk. Protopathic bias may also be relevant: because FAERS does not reliably capture the exact timing of drug initiation relative to symptom onset, it cannot be excluded that semaglutide was reported in some cases where early, unrecognized signs of an evolving pregnancy complication had already begun, potentially inflating the apparent association. Finally, our comparator groups did not include other GLP-1 receptor agonists specifically; we therefore cannot determine whether the signals observed here are specific to semaglutide or reflect a broader class effect, a question that would require a dedicated head-to-head comparison.

Furthermore, when reporting for a given drug is strongly concentrated within one or a few SOCs, the reporting proportion for all other SOCs is mechanically reduced, a phenomenon known as masking (or competition bias), which may account for some of the reduced RORs observed in our analysis independently of any true protective effect. Women exposed to semaglutide often have obesity and/or diabetes, conditions independently associated with adverse pregnancy outcomes; therefore, confounding by indication and underlying maternal risk factors cannot be ruled out. We partially addressed this through a secondary active-comparator analysis. Moreover, we performed a subgroup analysis stratifying semaglutide-exposed reports by indication (diabetes vs. obesity) and by formulation (injectable vs. oral); however, the comparator group was not itself restricted to indication-matched drugs (e.g., other antidiabetics or other anti-obesity agents), and subgroup sample sizes, particularly for the oral formulation, were limited, restricting the precision of these estimates.

## 4. Materials and Methods

### 4.1. Data Source

We retrieved the American standard code for information interchange (ASCII) data files from the FDA Adverse Event Reporting System (FAERS) database [52]. Specifically, we downloaded the Individual Case Safety Reports (ICSRs) from the FAERS database covering the period from the first quarter of 2017 to the second quarter of 2025. We selected this period for our analysis because semaglutide received FDA approval in 2017. Each file follows a specific structure and includes multiple datasets: “DEMO” (demographic information), “DRUG” (suspected/concomitant drug information), “REAC” (description of the reported adverse events), “OUTC” (outcome information), “THER” (treatment data), “INDI” (indication of use of the drugs). Each year in the FAERS database is divided into four quarters, resulting in a total of 216 ASCII data files for this period. These files were gathered to conduct comprehensive data mining and analysis. Both adverse events and the drug indication are coded in the Preferred Term (PT) level medical terminology, according to the Medical Dictionary for Regulatory Activities (MedDRA), version 28.0 [32].

### 4.2. Data Mining

To ensure the quality and integrity of the data, we performed data cleaning, which included a two-step deduplication process: (i) in the first step, we followed the FDA-recommended methods to remove duplicate records; specifically, we removed duplicate records from the “DEMO” dataset by deleting the earliest “FDA_DT” (i.e., the date FDA received the case) column when the “CASEID” (i.e., the number for identifying a FAERS case) column was identical; (ii) in the second step, we applied additional deduplication criteria by considering the following key variables: sex and age of the patient, the country from which the report was submitted, the date when the adverse event occurred, the name of the suspected or interacting or concomitant drug, its indication, the start and the end date of the drug therapy. Furthermore, we used the DiAna dictionary to map the drug names to their active ingredients and subsequently categorized these active ingredients according to the ATC classification [53].

### 4.3. Study Population

Our analysis focused on evaluating the safety of semaglutide following unintended exposure during pregnancy, analyzing the ICSRs that listed the mother as the patient and semaglutide as the suspected drug. To identify relevant cases, we filtered the initial dataset based on the following PTs: “Exposure during pregnancy”, “Maternal exposure during pregnancy”, “Pregnancy”, “Second trimester pregnancy”, and “Third trimester pregnancy”. Additionally, we filtered the dataset using Standardized MedDRA Queries (SMQs) related to adverse events during pregnancy, including “Foetal disorders,” “Neonatal disorders,” “Pregnancy, labour and delivery complications and risk factors,” and “Termination of pregnancy and risk of abortion”. Because FAERS does not include a structured field for conception date or last menstrual period, the exact timing of semaglutide exposure relative to conception could not be verified from the source data; cases were classified as pregnancy-exposed based solely on the PT/SMQ terms described above, irrespective of whether the reported information indicated exposure strictly during pregnancy or shortly before conception was recognized. Records with missing or incomplete information on gestational timing were retained in the primary analysis rather than excluded, in order to avoid selection bias toward better-documented reports.

### 4.4. Descriptive Analysis

A descriptive analysis was conducted to summarize the demographic characteristics of the ICSRs and the main features of the reported adverse events. In particular, we providing information about reporting year, source qualification, and the country for regulatory purposes, baseline demographic characteristics of patients (age and sex), suspect drugs (indication of use), and ADRs (number, and frequencies by SOC). We assessed the frequency of adverse events by System Organ Class (SOC). Additionally, we placed a particular focus on pregnancy outcomes and fetal/neonatal disorders.

### 4.5. Disproportionality Analysis

To evaluate the relationship between specific adverse events and semaglutide, a disproportional analysis was conducted. This analysis aimed to estimate the odds of reporting certain adverse events (categorized by SOC) associated with semaglutide in comparison to other drugs in pregnant women. The reporting odds ratio (ROR) was calculated with a 95% confidence interval (95% CI) using the following formula:

$$ROR=\frac{a/b}{c/d},\;95\%\;CI=exp\left\{\ln(ROR)\left.\pm 1.96\sqrt{\frac{1}{a}+\frac{1}{b}+\frac{1}{c}+\frac{1}{d}}\right\}\right.$$

where (a) was the number of events related to a specific SOC when semaglutide was the suspected drug, (b) was the number of events not related to that specific SOC when semaglutide was the suspected drug, (c) was the number of events related to a specific SOC when other drugs were the suspected drug, and (d) was the number of events not related to that specific SOC when other drugs were the suspected drug. We did not calculate the ROR when (a) or (c) were less than three. A *p*-value of < 0.05 was used for statistical significance.

Moreover, we focused our analysis on the pregnancy-related adverse events, determining whether pregnant women exhibit a lower/higher probability of reporting pregnancy-related adverse events when using semaglutide compared to other drugs. In this case, (a) was the number of adverse pregnancy outcomes and fetal/neonatal disorders associated with semaglutide as a suspected drug, (b) was the number of adverse events not related to adverse pregnancy outcomes and fetal/neonatal disorders associated with semaglutide as a suspected drug, (c) was the number of adverse pregnancy outcomes and fetal/neonatal disorders associated with other drugs as suspected drugs, and (d) was the number of adverse events not related to adverse pregnancy outcomes and fetal/neonatal disorders associated with other drugs as suspected drugs.

To assess whether the disproportionality signal was consistent across formulation and indication, we performed two subgroup analyses. Semaglutide-exposed reports were stratified, when the formulation could be identified from the reported brand name, into injectable (Ozempic^®^, Wegovy^®^) and oral (Rybelsus^®^) subgroups; reports listing only the generic name “semaglutide” were excluded from this stratification. Reports were also stratified by indication (diabetes vs. obesity/weight-control), based on the indication linked to the semaglutide drug record itself via the drug sequence number. In both stratifications, the comparator group was unchanged from the main analysis, and RORs with 95% CIs were calculated as described above at the SOC and SMQ level. Given that the SMQ “Termination of pregnancy and risk of abortion” aggregates PTs with heterogeneous clinical meaning, we further examined this SMQ by classifying its constituent PTs into three categories: spontaneous pregnancy loss (e.g., “Abortion spontaneous,” “Abortion missed,” “Anembryonic gestation”), elective/induced termination (e.g., “Abortion induced,” “Selective abortion”), and terms for which MedDRA coding does not specify the type (e.g., “Abortion,” “Abortion complete/incomplete”). RORs and 95% CIs were calculated separately for each of the three categories against the same comparator used for the main analysis.

As a secondary analysis, we additionally benchmarked semaglutide against an indication-matched active comparator, consisting of pregnancy reports in which a drug other than semaglutide carried a diabetes or obesity/weight-control indication. This comparator was defined by indication rather than by a curated drug-name list. Because indication fields in FAERS are frequently missing, this active-comparator population likely under-ascertains the true population of diabetic/obese pregnant patients receiving other therapies. RORs and 95% CIs versus this secondary comparator were calculated identically to the primary analysis, at both the SOC and SMQ level.

### 4.6. Sensitivity Analyses

To assess the robustness of the main findings, three sensitivity analyses were performed. First, the disproportionality analyses were repeated after restricting the dataset to ICSRs in which semaglutide was classified as the primary suspect (PS) drug, thereby excluding reports in which semaglutide was recorded as a secondary suspect. Second, analyses were repeated after excluding ICSRs reporting concomitant exposure to medications with established teratogenic potential, in order to minimize potential confounding related to co-medications. Teratogenic medications were identified according to a previously published classification based on the Teratogen Information System (TERIS) and Clinical Pharmacology^®^ monographs, including drugs with known teratogenic risk [54]. Third, analyses were restricted to reports submitted by physicians to evaluate the consistency of the findings in reports expected to have higher clinical reliability. The same statistical approach adopted for the primary analysis was applied to each sensitivity analysis, and the corresponding reporting RORs with 95% CIs were calculated.

All data management, data cleaning and statistical analyses were performed using R software (version 4.5.1; R Foundation for Statistical Computing, Vienna, Austria).

## 5. Conclusions

Our analysis provides an overview of pregnancy-related adverse event reports associated with unintended semaglutide exposure during pregnancy retrieved from a large spontaneous reporting system. We specifically focused on maternal and fetal outcomes, as well as on reporting patterns identified through disproportionality analysis, in order to explore potential safety signals in a population systematically excluded from clinical trials. Overall, the findings suggest heterogeneous reporting patterns. Reporting odds were increased for “Pregnancy, puerperium and perinatal conditions,” “Injury, poisoning and procedural complications,” and the SMQ “Termination of pregnancy and risk of abortion”—driven predominantly by spontaneous pregnancy loss—while “Neonatal disorders” showed significantly lower reporting. These observations should be interpreted cautiously, as they may reflect reporting dynamics, underlying maternal metabolic conditions, or reproductive decision-making processes rather than a direct pharmacological effect.

Given the intrinsic limitations of spontaneous reporting data, including underreporting, lack of denominator information, and the inability to control for confounding by indication, no causal inferences can be drawn. The present findings are intended to identify potential safety concerns for further evaluation and should not be interpreted as evidence of causality. Nevertheless, in the context of the rapidly expanding use of semaglutide among women of reproductive age, our findings highlight the need for well-designed prospective studies and pregnancy registries to better characterize the safety profile of inadvertent early pregnancy exposure to semaglutide. Specifically, prospective pregnancy exposure registries with structured collection of gestational timing, dosing, and outcomes would directly address the inability of spontaneous reporting systems to capture exposure timing. Large-scale, EHR- or claims-based cohort studies using active comparators (e.g., insulin or other antidiabetic/anti-obesity agents), similar in design to existing multi-country studies of other antidiabetics in pregnancy, would allow more rigorous control for confounding by indication than disproportionality analysis permits. From a clinical perspective, our findings support discontinuing semaglutide well before conception and reinforce effective contraceptive counselling during treatment. For inadvertent early-pregnancy exposure, they do not support an increased risk of major congenital anomalies and should not prompt pregnancy termination on teratogenic grounds; prompt discontinuation, transition to an alternative therapy, closer obstetric monitoring, and reporting to pregnancy exposure registries remain reasonable pending further prospective data.

## Figures and Tables

**Table 1:** Characteristics of individual case safety reports (ICSRs) with semaglutide in pregnant patients from the FAERS database (Q1 2018 to Q2 2025).

Characteristics	No. of Cases (%)
**Total**	249 (100.0)
**Indication of use**	
Diabetes	113 (45.1)
Obesity	120 (48.2)
Product used for unknown indication	16 (6.4)
**Formulation**	
Injectable	211 (84.8)
Oral	11 (4.4)
Not specified	27 (10.8)
Trimester of exposure	
Not specified	249 (100.0)
**Reported Country**	
United States of America	105 (42.2)
Denmark	18 (7.2)
Canada	15 (6.0)
Great Britain	15 (6.0)
France	9 (3.6)
Norway	8 (3.2)
Australia	7 (2.8)
Switzerland	7 (2.8)
Belgium	6 (2.4)
Brazil	6 (2.4)
China	6 (2.4)
**Source qualification**	
Consumer	104 (41.8)
Physician	86 (34.5)
Pharmacist	14 (5.6)
Other health-professional	2 (0.8)
Not available	43 (17.3)
**Median age** (**IQR**)	34 (29–39)
**Reporting year**	
2018	3 (1.2)
2019	1 (0.4)
2020	5 (2.0)
2021	15 (6.0)
2022	21 (8.4)
2023	66 (26.5)
2024	83 (33.3)
2025	55 (22.1)
***n*. of adverse events** (**median number per ICSR, IQR**)	3 (2–4)

**Table 2:** Frequency of suspected adverse drug reactions included in the standardized MedDRA Queries (SMQs) “Pregnancy, labour and delivery complications”, “Foetal disorders”, “Neonatal disorders”, “Termination of pregnancy”. Preferred terms reported in ≤2 cases are omitted from this table and reported in Table S1 (Supplementary Materials).

Pregnancy-Related Adverse Events (Preferred Terms)	*n*. of Events	Percentage
Maternal exposure during pregnancy	162	38.2%
Abortion spontaneous	88	20.8%
Foetal exposure during pregnancy	38	9.0%
Exposure during pregnancy	16	3.8%
Abortion induced	10	2.4%
Premature baby	8	1.9%
Premature delivery	8	1.9%
Ectopic pregnancy	7	1.7%
Morning sickness	6	1.4%
Abortion missed	5	1.2%
Abortion	4	0.9%
Foetal death	4	0.9%
Foetal growth restriction	4	0.9%
Gestational diabetes	4	0.9%
Polyhydramnios	4	0.9%
Oligohydramnios	3	0.7%
Omphalitis	3	0.7%
Pre-eclampsia	3	0.7%
Stillbirth	3	0.7%

**Table 3:** Comparative analysis of adverse events in pregnant women, evaluating the odds of reported events associated with semaglutide compared with other medications.

System Organ Class	Semaglutide in Pregnant Patients vs. Other Drugs in Pregnant Patients ROR (95% CI)
Blood and lymphatic system disorders	0.58 (0.26–1.31)
Cardiac disorders	0.51 (0.26–1.03)
Congenital, familial and genetic disorders	1.22 (0.91–1.62)
Ear and labyrinth disorders	(less than 3 cases)
Endocrine disorders	(less than 3 cases)
Eye disorders	(less than 3 cases)
Gastrointestinal disorders	1.72 (1.38–2.15)
General disorders and administration site conditions	0.40 (0.29–0.54)
Hepatobiliary disorders	(less than 3 cases)
Immune system disorders	(less than 3 cases)
Infections and infestations	0.78 (0.55–1.12)
Injury, poisoning and procedural complications	1.93 (1.70–2.20)
Investigations	0.67 (0.47–0.97)
Metabolism and nutrition disorders	1.25 (0.83–1.87)
Musculoskeletal and connective tissue disorders	0.43 (0.28–0.66)
Neoplasms benign, malignant and unspecified	(less than 3 cases)
Nervous system disorders	0.66 (0.47–0.93)
Pregnancy, puerperium and perinatal conditions	1.59 (1.37–1.86)
Product issues	(less than 3 cases)
Psychiatric disorders	0.30 (0.16–0.54)
Renal and urinary disorders	(less than 3 cases)
Reproductive system and breast disorders	0.54 (0.26–1.13)
Respiratory, thoracic and mediastinal disorders	0.71 (0.47–1.07)
Skin and subcutaneous tissue disorders	0.34 (0.18–0.64)
Social circumstances	(less than 3 cases)
Surgical and medical procedures	0.97 (0.60–1.57)
Vascular disorders	(less than 3 cases)

**Table 4:** Comparative analysis of pregnancy-related adverse events in pregnant women, evaluating the odds of reported events associated with semaglutide compared with other medications. The adverse events were categorized as Standardized MedDRA Query (SMQ).

Standardized MedDRA Query	Semaglutide in Pregnant Patients vs. Other Drugs in Pregnant Patients ROR (95% CI)
Foetal disorders	1.24 (0.82–1.87)
Neonatal disorders	0.37 (0.24–0.56)
Pregnancy, labour and delivery complications and risk factors (excl abortions and stillbirth)	1.42 (1.24–1.63)
Termination of pregnancy and risk of abortion	3.71 (3.07–4.48)

**Table 5:** Comparative analysis of adverse events coded as System Organ Class in pregnant women, evaluating the odds of reported events associated with semaglutide compared with other medications stratified by formulation (injectable and oral) and indication (diabetes and obesity/weight-control). Comparator: other drugs in pregnant patients.

System Organ Class	Injectable	Oral	Diabetes Indication	Obesity/Weight-Control Indication
Blood and lymphatic system disorders	(less than 3 cases)	(less than 3 cases)	(less than 3 cases)	(less than 3 cases)
Cardiac disorders	0.48 (0.22–1.08)	(less than 3 cases)	1.32 (0.54–3.20)	(less than 3 cases)
Congenital, familial and genetic disorders	1.35 (0.99–1.84)	(less than 3 cases)	1.43 (0.83–2.45)	1.73 (1.12–2.67)
Ear and labyrinth disorders	(less than 3 cases)	(less than 3 cases)	(less than 3 cases)	(less than 3 cases)
Endocrine disorders	(less than 3 cases)	(less than 3 cases)	(less than 3 cases)	(less than 3 cases)
Eye disorders	(less than 3 cases)	(less than 3 cases)	(less than 3 cases)	(less than 3 cases)
Gastrointestinal disorders	1.40 (1.07–1.83)	3.35 (1.40–8.00)	1.96 (1.28–2.98)	1.13 (0.71–1.80)
General disorders and administration site conditions	0.31 (0.21–0.45)	(less than 3 cases)	0.46 (0.26–0.83)	0.14 (0.06–0.34)
Hepatobiliary disorders	(less than 3 cases)	(less than 3 cases)	(less than 3 cases)	(less than 3 cases)
Immune system disorders	(less than 3 cases)	(less than 3 cases)	(less than 3 cases)	(less than 3 cases)
Infections and infestations	0.47 (0.28–0.78)	(less than 3 cases)	0.51 (0.21–1.23)	(less than 3 cases)
Injury, poisoning and procedural complications	2.25 (1.95–2.60)	1.18 (0.58–2.44)	1.34 (1.02–1.77)	3.10 (2.48–3.86)
Investigations	0.62 (0.41–0.95)	(less than 3 cases)	1.12 (0.63–2.00)	0.27 (0.10–0.73)
Metabolism and nutrition disorders	1.31 (0.84–2.04)	(less than 3 cases)	1.93 (0.99–3.75)	0.96 (0.43–2.16)
Musculoskeletal and connective tissue disorders	0.35 (0.20–0.59)	(less than 3 cases)	0.73 (0.38–1.43)	(less than 3 cases)
Neoplasms benign, malignant and unspecified	(less than 3 cases)	(less than 3 cases)	(less than 3 cases)	(less than 3 cases)
Nervous system disorders	0.71 (0.49–1.03)	(less than 3 cases)	0.47 (0.21–1.05)	0.18 (0.06–0.55)
Pregnancy, puerperium and perinatal conditions	1.76 (1.49–2.08)	2.45 (1.21–4.93)	1.60 (1.17–2.17)	2.25 (1.76–2.88)
Product issues	(less than 3 cases)	(less than 3 cases)	(less than 3 cases)	(less than 3 cases)
Psychiatric disorders	0.37 (0.21–0.68)	(less than 3 cases)	(less than 3 cases)	(less than 3 cases)
Renal and urinary disorders	(less than 3 cases)	(less than 3 cases)	(less than 3 cases)	(less than 3 cases)
Reproductive system and breast disorders	0.39 (0.14–1.03)	(less than 3 cases)	0.94 (0.30–2.94)	0.71 (0.23–2.23)
Respiratory, thoracic and mediastinal disorders	0.31 (0.15–0.61)	(less than 3 cases)	1.01 (0.50–2.05)	(less than 3 cases)
Skin and subcutaneous tissue disorders	0.34 (0.17–0.69)	(less than 3 cases)	0.42 (0.13–1.31)	(less than 3 cases)
Social circumstances	(less than 3 cases)	(less than 3 cases)	(less than 3 cases)	(less than 3 cases)
Surgical and medical procedures	1.01 (0.59–1.71)	4.76 (1.46–15.49)	1.41 (0.63–3.16)	1.07 (0.47–2.39)
Vascular disorders	(less than 3 cases)	(less than 3 cases)	(less than 3 cases)	(less than 3 cases)

**Table 6:** Comparative analysis of adverse events coded as Standardized MedDRA Query in pregnant women, evaluating the odds of reported events associated with semaglutide compared with other medications stratified by formulation (injectable and oral) and indication (diabetes and obesity/weight-control). Comparator: other drugs in pregnant patients.

Standardized MedDRA Query	Injectable	Oral	Diabetes Indication	Obesity/Weight-Control Indication
Foetal disorders	1.35 (0.87–2.11)	(fewer than 3 cases)	2.46 (1.34–4.50)	1.16 (0.55–2.46)
Neonatal disorders	0.29 (0.17–0.50)	(fewer than 3 cases)	0.91 (0.52–1.60)	0.21 (0.08–0.55)
Pregnancy labour and delivery complications and risk factors	1.51 (1.30–1.76)	2.02 (1.04–3.90)	1.32 (0.99–1.75)	2.03 (1.62–2.55)
Termination of pregnancy and risk of abortion	4.34 (3.56–5.30)	3.04 (1.08–8.58)	2.19 (1.37–3.51)	5.39 (4.03–7.21)

**Table 7:** Comparative disproportionality analysis of adverse events associated with semaglutide in pregnant patients by System Organ Class and by Standardized MedDRA Query: main analysis (vs. all other drugs in pregnant patients) versus a secondary, indication-matched active comparator (other antidiabetic/anti-obesity drugs in pregnant patients).

**System Organ Class**	**ROR (95% CI)**
Blood and lymphatic system disorders	0.54 (0.24–1.23)
Cardiac disorders	0.75 (0.37–1.54)
Congenital, familial and genetic disorders	0.74 (0.55–0.99)
Ear and labyrinth disorders	(fewer than 3 cases)
Endocrine disorders	(fewer than 3 cases)
Eye disorders	(fewer than 3 cases)
Gastrointestinal disorders	2.59 (2.05–3.28)
General disorders and administration site conditions	0.85 (0.62–1.17)
Hepatobiliary disorders	(fewer than 3 cases)
Immune system disorders	(fewer than 3 cases)
Infections and infestations	1.21 (0.84–1.76)
Injury, poisoning and procedural complications	1.50 (1.31–1.71)
Investigations	0.60 (0.41–0.87)
Metabolism and nutrition disorders	0.46 (0.31–0.70)
Musculoskeletal and connective tissue disorders	1.51 (0.97–2.35)
Neoplasms benign, malignant and unspecified	(fewer than 3 cases)
Nervous system disorders	0.81 (0.57–1.15)
Pregnancy, puerperium and perinatal conditions	1.01 (0.86–1.18)
Product issues	(fewer than 3 cases)
Psychiatric disorders	0.53 (0.29–0.97)
Renal and urinary disorders	(fewer than 3 cases)
Reproductive system and breast disorders	0.68 (0.32–1.45)
Respiratory, thoracic and mediastinal disorders	0.81 (0.53–1.23)
Skin and subcutaneous tissue disorders	0.65 (0.34–1.22)
Social circumstances	(fewer than 3 cases)
Surgical and medical procedures	1.20 (0.73–1.97)
Vascular disorders	(fewer than 3 cases)
**Standardized MedDRA Query**	**ROR** (**95% CI**)
Foetal disorders	0.66 (0.43–1.00)
Neonatal disorders	0.22 (0.14–0.33)
Pregnancy, labour and delivery complications and risk factors	0.78 (0.68–0.90)
Termination of pregnancy and risk of abortion	4.19 (3.41–5.17)

## Data Availability

The original data presented in the study are openly available in FAERS Quarterly Data Files Documentation at https://www.fda.gov/drugs/fda-adverse-event-monitoring-system-aems/faers-quarterly-data-files-documentation, accessed on 2 August 2026.

## Supplementary Materials

The following supporting information can be downloaded at: https://www.mdpi.com/article/10.3390/ph19081249/s1, Table S1. Preferred terms (PTs) associated with semaglutide-exposed pregnancies reported in one or two cases only, omitted from Table 2 for clarity.

## Abbreviations

The following abbreviations are used in this manuscript:

ADR	Adverse Drug Reaction
AE	Adverse Event
FAERS	FDA Adverse Event Reporting System
GLP-1 RAs	Glucagon-like peptide-1 receptor agonists
ICSR	Individual case safety report
MedDRA	Medical Dictionary for Regulatory Activities
PT	Preferred Term
SmPC	Summary of Product Characteristics
