# Supplementary material for: Semaglutide Exposure During Pregnancy: Results from the FAERS Database
Source: Pharmaceuticals (Basel). 2026 Aug 8;19(8):1249. doi: 10.3390/ph19081249 (PMC13516160; doi:10.3390/ph19081249)
Supplement: Supplementary file 1 [file pharmaceuticals-19-01249-s001.zip › pharmaceuticals-4446549-supplementary.pdf]

Table S1. Preferred terms (PTs) associated with semaglutide-exposed pregnancies reported in one or two cases only, omitted from Table 2 for clarity.

Pregnancy-related adverse events (preferred terms)	N. of events	Percentage
Caesarean section	2	0.5%
Feeding intolerance	2	0.5%
Foetal heart rate abnormal	2	0.5%
Hypoglycaemia neonatal	2	0.5%
Low birth weight baby	2	0.5%
Maternal drugs affecting foetus	2	0.5%
Placental insufficiency	2	0.5%
Amniotic fluid volume decreased	1	0.2%
Anembryonic gestation	1	0.2%
Antiphospholipid syndrome	1	0.2%
Chloasma	1	0.2%
Developmental hip dysplasia	1	0.2%
Drug exposure before pregnancy	1	0.2%
Foetal disorder	1	0.2%
Foetal growth abnormality	1	0.2%
Foetal macrosomia	1	0.2%
Foetal malformation	1	0.2%
Foetal vascular malperfusion	1	0.2%
Haemorrhage in pregnancy	1	0.2%
High-pitched crying	1	0.2%
Hyperemesis gravidarum	1	0.2%
Maternal condition affecting foetus	1	0.2%
Maternal exposure before pregnancy	1	0.2%
Maternal exposure timing unspecified	1	0.2%
Neonatal hyperglycaemia	1	0.2%
Neonatal respiratory failure	1	0.2%
Perineal swelling	1	0.2%
Postpartum haemorrhage	1	0.2%
Premature labour	1	0.2%
Preterm premature rupture of membranes	1	0.2%
Shoulder dystocia	1	0.2%
Small for dates baby	1	0.2%
Subchorionic haemorrhage	1	0.2%
Umbilical discharge	1	0.2%
Vacuum extractor delivery	1	0.2%
Vanishing twin syndrome	1	0.2%
Vulvovaginal injury	1	0.2%
